# Two-Week Exclusive Supplementation of Modified Ketogenic Nutrition Drink Reserves Lean Body Mass and Improves Blood Lipid Profile in Obese Adults: A Randomized Clinical Trial

**DOI:** 10.3390/nu10121895

**Published:** 2018-12-03

**Authors:** Hae-Ryeon Choi, Jinmin Kim, Hyojung Lim, Yoo Kyoung Park

**Affiliations:** 1 Department of Medical Nutrition, Graduate School of East-West Medical Science, Kyung Hee University, Yongin, Gyeonggi-do 17104, Korea; hrchoi92@naver.com; 2 Nutritional Product R&D team, Maeil Innovation Center, Maeil Dairies Co., Ltd., Pyeongtaek, Gyeonggi-do 17714, Korea; kim0521@maeil.com; 3 MDwell Inc., Seoul 06170, Korea; hjlim@daewoong.co.kr

**Keywords:** obesity, ketogenic diet, weight loss, nutrition drink, food for special dietary use

## Abstract

The ketogenic diet has long been recommended in patients with neurological disorders, and its protective effects on the cardiovascular system are of growing research interest. This study aimed to investigate the effects of two-week of low-calorie ketogenic nutrition drinks in obese adults. Subjects were randomized to consume drinks either a ketone-to-non-ketone ratio of 4:1 (KD 4:1), a drink partially complemented with protein at 1.7:1 (KD 1.7:1), or a balanced nutrition drink (BD). Changes in body weight, body composition, blood lipid profile, and blood ketone bodies were investigated. Blood ketone bodies were induced and maintained in the group that consumed both 4:1 and 1.7:1 ketogenic drinks (*p* < 0.001). Body weight and body fat mass significantly declined in all groups between 0 and 1 week and between 1 and 2 weeks (*p* < 0.05), while skeletal muscle mass remained unchanged only in the KD 1.7:1 group (*p* > 0.05). The blood lipid profile improved, appetite was reduced, and fullness was maintained in the two ketogenic drink groups. This study indicates the possibility for the development of obesity treatments based on ketogenic nutrition drinks even with a moderate ketogenic ratio of 1.7:1, as well as adjuvant therapies based on ketosis induction and maintenance for the treatment of other diseases and health conditions.

## 1. Introduction

According to the World Health Organization (WHO), 39% of the adult population is overweight, of which 13% is classified as obese [1]. In Korea also, one out of three adults is obese, and the prevalence of obesity has increased steadily from 26.0% in 1998 to 34.8% in 2016 [2]. Several trials have been conducted on the effective prevention and management of obesity and development of obesity treatments [2,3,4,5].

Recently, there has been keen interest in low-carbohydrate, high-fat diets, which strictly limit carbohydrate consumption and allow unlimited consumption of high-fat foods, such as pork belly, butter, and cheese, and their effectiveness in reducing weight and improving blood lipid profiles. Until recently, high-fat diets had been believed to increase the risk of obesity and cardiovascular disease, and thus calorie-restricted, low-fat diets have been recommended for preventing and treating obesity [6]. Although the rate of fat intake has decreased steadily in the United States since the 1970s, the prevalence of obesity has continued to rise, leading many to question high fat intake as a risk factor for weight gain [7,8]. During the same period, the intake of refined carbohydrates has increased, leading to greater attention being paid to high-carbohydrate diets as a cause of the increased prevalence of obesity and growing interest in low-carbohydrate diets [9,10].

As low-carbohydrate, high-fat diets consistently limit the intake of carbohydrates, the body is forced to use glycogen to maintain blood glucose levels. When glycogen levels decrease, the body then uses ketone bodies generated though lipid degradation as its main source of energy. Therefore, diets that are low in carbohydrates and high in fat are called “ketogenic diets” [11]. The typical ratio of fat to carbohydrates and protein in a ketogenic diet is 3:1 or 4:1 [12]. 

Although the ketogenic diet was initially developed as a treatment for incurable pediatric epilepsy, recent studies have found that it is an effective treatment for a variety of conditions. In particular, it has been shown to reduce the risk factors for obesity, diabetes, and cardiovascular disease, and several studies have recently been conducted on its therapeutic effects on acne, neurological disorders, cancer, and polycystic ovarian syndrome [13]. According to studies on the relationship between the ketogenic diet and risk factors for obesity and cardiovascular disease, the diet has been found to be effective in reducing weight through fat-free mass maintenance, body fat reduction, body water reduction [14,15,16], and improving the blood lipid profile (total cholesterol, triglycerides, and LDL and HDL cholesterol) [17,18,19]. 

As there is no standard ratio of macronutrients for low-carbohydrate, high-fat diet therapies, various ratios have been used in different studies [20]. Due to the lack of studies that have been done on the effects of the ketogenic ratio on weight loss and blood lipid profile improvement among obese adults, more studies will need to be conducted in the future to investigate the effects of the diet’s ketogenic ratio and macronutrient energy ratio (carbohydrates, protein, and fat) on obese adults.

For safe and effective weight loss and maintenance, diet compliance is crucial. In fact, compliance has been shown to be more important than the type of diet therapy [21]. Culture and tradition, dietary habits, food preferences, and medical history should all be taken into account when seeking to increase compliance with diet therapy for the treatment of obesity. Considering that carbohydrates account for up to 63% of Koreans’ diet [22], it is generally believed to be difficult for Asian people to maintain a low-carbohydrate, high-fat diet for a long period of time. 

In light of these circumstances, in this study, changes in body weight, body composition, blood lipid profiles, and blood ketone bodies were investigated in healthy obese adults after a two-week period in which they consumed a ketogenic nutrition drink exclusively, with a ketone-to-non-ketone ratio of 4:1 (KD 4:1), the drink partially complemented with protein to yield a ketone-to-non-ketone ratio of 1.7:1 (KD 1.7:1), or a balanced nutrition drink (BD) with an energy ratio based on Korean nutritional intake standard.

## 2. Materials and Methods 

### 2.1. Study Period and Participants

This study was conducted following the deliberation and approval of the Institutional Review Board (IRB) of Kyung Hee University (KHU IRB No. KHSIRB-17-028). The subjects of the study were healthy adults ranging in age from 19 to 49 years and were recruited between September and December of 2017. All selected subjects had experienced no weight change within the three months prior to the study and had a body mass index (BMI) of over 25 kg/m^2^. The exclusion criteria were: (1) Men with a body fat ratio of less than 12% and women with a body fat ratio of less than 30%; (2) individuals who had engaged in a dietary regimen, taken diet pills, or had surgery for weight loss within the six months prior to the study; (3) individuals who had become obese due to endocrine disorders or medication, with severe depression or other mental disorders, alcoholism, and/or pathophysiological risk factors, including respiratory diseases, diabetes/insulin treatment, cardiovascular disease, or kidney disorders; and (4) individuals taking regular medication; (5) pregnant or breastfeeding. Before starting the study, applicants were fully informed of the purpose, content, and procedure of the study. Consent was received voluntarily. Of the 48 subjects who applied to participate in the study, two were excluded, leaving a total of 46 study subjects. Through stratified randomization, the subjects were assigned to one of three groups with equal gender distribution: Ketogenic nutrition drink 4:1 group (KD 4:1 group, with a ketone-to-non-ketone ratio (fat-to-carbohydrate + protein) of 4:1), modified ketogenic nutrition drink 1.7:1 group (KD 1.7:1 group, with a ketone-to-non-ketone ratio of 1.7:1), and the balanced nutrition drink group (BD group). The subjects received liquid-type nutrition drinks with nutrient profiles designed especially for their group. The C:P:F profile (% of kcal) of the drink for the KD 4:1 group was 3:7:90, while those for the KD 1.7:1 and BD groups were 4:16:80 and 54:16:30, respectively (Table 1). 

### 2.2. Study Design

This study was a randomized nutritional intervention trial that was conducted over a period of two weeks with 46 subjects who received nutrition drinks assigned specifically to their given groups that were consumed as a meal substitute. Subjects’ physical activity was monitored to maintain stability. 

All measurement were performed three times: Before (0 week), during (1 week), and after (2 weeks) the intervention. The measurements included: Body weight, height, waist circumference, and hip circumference (anthropometric measurements); body water, protein, minerals, skeletal muscle mass, and body fat mass (body composition analysis); and blood lipid profile (total cholesterol, triglycerides, and LDL and HDL cholesterol) and ketone bodies, including total ketones, acetoacetate, and β-hydroxybutyric acid (blood collection and analysis). In addition, changes in physical activity and body symptoms were surveyed through questionnaires, and self-reported urinary ketone body values and nutrition drink consumption were examined.

### 2.3. Dietary Intervention and Monitoring of Physical Activity

#### 2.3.1. Dietary Intervention through Nutrition Drinks

**Figure 1:**
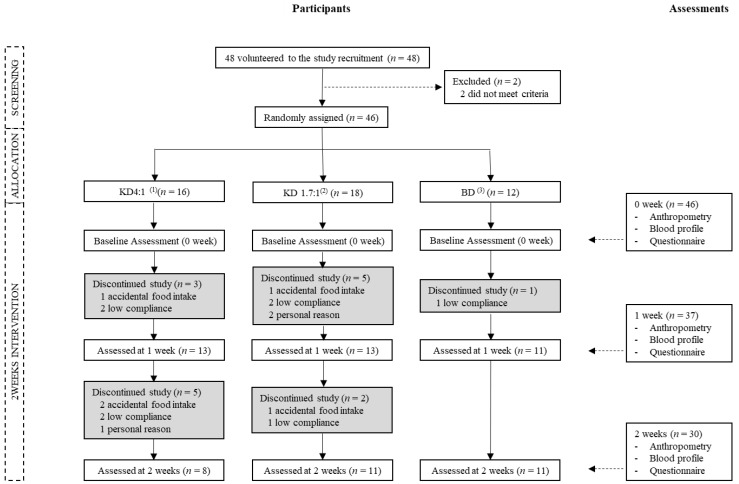
Diagram of study flow. ^(1)^ KD4:1 = Ketogenic nutrition drink 4:1 (Ketonina, Namyang Dairy Product Co Ltd., Seoul, Korea). ^(2)^ KD1.7:1 = Ketogenic nutrition drink 1.7:1 (Product for research, Maeil Dairies Co Ltd., Gyeonggi-doKorea). ^(3)^ BD = Balanced nutrition drink (Mediwell Nutty flavor, Maeil Dairies Co Ltd., Gyeonggi-do, Korea).

During the study intervention period, three subjects were dropped from the KD 4:1 group in the first week, along with five in the KD 1.7:1 group and one in the BD group. In the second week, five were dropped from the KD 4:1 group and two from the KD 1.7:1 group. Thus, a total of 30 subjects completed the study at the end of the second week, of which eight were in KD 4:1, 11 in KD 1.7:1, and 11 in BD. For the analysis, data were analyzed from a total of 37 subjects who completed at least the first week of the study, of which 13 were in KD 4:1, 13 in KD 1.7:1, and 11 in BD (Figure 1). During the study, subjects who ate foods other than provided nutrition drinks, did not keep their physical activity at their usual levels were also ruled out for analyses.

The daily energy requirements for all subjects were calculated according to the Harris–Benedict equation, taking into account each individual’s age, height, weight, and level of physical activity [23]. The recommended dietary intake was set at 70% of the daily requirement for weight loss, with the goal of generating a caloric deficit of approximately 600 kcal.

Subjects were instructed on the amount of nutrition drink to consume on a daily basis, as well as on the consumption method, issues requiring attention, and how to record their daily nutrition drink consumption.

#### 2.3.2. Monitoring of Physical Activity

All subjects were instructed to maintain their usual levels of physical activity during the study period. Any changes in physical activity were monitored using a self-assessment questionnaire that was selected from among the International Physical Activity Questionnaires (IPAQ) and conducted three times, once each in 0, 1, and 2 weeks.

### 2.4. Body Composition and Anthropometric Analysis

The body composition of subjects was measured after at least eight hours of fasting using Inbody 270 (Biospace, Seoul, Korea). Body weight, body water, protein, minerals, skeletal muscle mass, and body fat mass were measured three times, once each in 0, 1, and 2 weeks. For accurate measurement, subjects were lightly dressed with similar clothing for each visit, metal and accessories were removed, and measurements were made with no movement of the posture during the measurement. While height was self-reported by the subjects, waist circumference was measured at the point 2.5 cm above the navel, and hip circumference was measured at the highest point of the hip to the nearest 0.1 cm and averaged among two measurements. BMI was calculated by dividing each subject’s weight (kg) by the square of his or her height (m^2^).

### 2.5. Blood Analysis

Blood analysis was performed three times: Before (0 week), during (1 week), and after (2 weeks) the study. After an overnight fast of at least 8 h, blood samples of 5 mL of blood were collected in a non-heparinized container and coagulated for at least 30 min at room temperature and then centrifuged at 3500 rpm for 7 min. For each sample, 1 mL of separated serum was placed in a microtube and stored in a freezer. The frozen serum was analyzed by Green Cross Laboratories. Total cholesterol and triglyceride levels were analyzed using enzymatic colorimetric assay kits (CHOL2, Roche, Mannheim, Germany, and TRIGL, Roche, Mannheim, Germany), and LDL and HDL cholesterol were analyzed using homogeneous enzymatic colorimetric assay kits (LDL-Cholesterol Gen. 3, Roche, Germany, and HDL-C.plus Gen. 3, respectively). Finally, ketone bodies (total ketone, acetoacetate, and β-hydroxybutyric acid levels) were analyzed using a gas chromatography/mass spectrometry (GC/MS) kit (Green Cross Laboratories, Seoul, Korea).

### 2.6. Survey of Physical Symptoms

To examine the symptoms and difficulties that the subjects experienced during the study period, a survey composed of a total of 15 questions selected in reference to symptoms described in previous studies was conducted [14,15]. The survey was conducted in 0, 1, and 2 weeks, and the responses were scored on a five-point Likert scale (strongly disagree = 1, disagree = 2, neutral = 3, agree = 4, strongly agree = 5).

### 2.7. Urinary Ketone Bodies

To check the production and maintenance of ketone bodies, and to make sure the subjects ate other foods besides nutrition drinks, the subjects were instructed to measure their urinary ketone bodies at least three times a day (morning, noon, and evening) using a simple urinary ketone body test strip that changes color depending on the concentration of urinary ketone bodies (Uriscan Ketone Strip, YD Diagnostics, Yongin, Korea). The subjects exposed the strips to urine, waited one minute, compared its color with the color chart included in the package, and then indicated the result on the urinary ketone body form (negative, ±5, +10, ++50, or +++100).

### 2.8. Statistical Analysis

Data were analyzed using Version 23.0 of the Statistical Package for the Social Sciences (SPSS, IBM, Seoul, South Korea), and continuous variables were expressed as mean and standard deviation (SD) values and discrete variables as *N* (%). Changes in the values measured before, during, and after the study were analyzed using the paired *t*-test. while individual analyses for significant items were conducted using the paired *t*-test and Wilcoxon signed-rank test. The significance between discrete frequencies was analyzed using the chi-square tests, while the differences among the three subject groups were analyzed using one-way ANOVA and non-parametric Kruskal–Wallis tests. All results from statistical analysis were tested for significance at *p* < 0.05.

## 3. Results

### 3.1. General Characteristics

Among the three groups of study participants, there were no significant differences in age, sex, weight, body water, protein, minerals, body weight, skeletal muscle mass, body fat mass, BMI, waist and hip circumferences, total cholesterol, triglycerides, and LDL and HDL cholesterol (Table 2) at baseline.

### 3.2. Average Intake During the Study Period

During the two-week intervention period, daily energy intake of the subjects was 1159.1 ± 416 kcal for the KD 4:1 group, 1280.8 ± 262.2 kcal for the KD 1.7:1 group, and 1357.1 ± 292.5 kcal for the BD group. The difference between the daily energy requirement and actual energy intake during the study period was 799.7 ± 373.2 kcal for the KD 4:1 group, 743.8 ± 157.5 kcal for the KD 1.7:1 group, and 716.2 ± 94.4 kcal for the BD group. In this regard, no significant differences were found among the three groups. 

Carbohydrate, fiber, protein, and fat intake were significantly different among the three groups. In particular, the saturated fat intake was 24.1 ± 8.7 g for the KD 4:1 group, 0.0 ± 0.0 g for the KD 1.7:1 group, and 13.6 ± 2.9 g for the BD group, showing significant variation among the groups (Table 3).

### 3.3. Changes in Body Composition and Anthropometric Measurements

In all groups, significant differences in body water, protein, minerals, body weight, skeletal muscle mass, body fat mass, BMI, waist circumference, and hip circumference were found between 0 week and 2 weeks.

All groups showed a significant decrease in body water and minerals between 0 week and 1 week but no significant change from 1 week to 2 weeks. Protein and skeletal muscle mass decreased significantly between 0 and 1 week and between 1 and 2 weeks in the KD 4:1 and BD groups, but showed no significant change in the KD 1.7:1 group. In all groups, body weight, body fat mass, and BMI recorded significant decreases between 0 and 1 week and between 1 and 2 weeks. 

In all groups, waist and hip circumference decreased significantly between 0 week and 1 week (Table 4).

### 3.4. Changes in Blood Lipids

Total cholesterol showed no significant change between 0 week and 2 week in the KD 4:1 group but decreased significantly in the KD 1.7:1 and BD groups. In all groups, no significant differences in total cholesterol were observed between 0 week and 1 week, but a significant decrease was recorded between 1 week and 2 weeks. In all groups, triglycerides recorded no significant change across all weeks of the intervention period. LDL cholesterol showed no significant change between 0 week and 2 weeks in the KD 4:1 group but decreased significantly in the KD 1.7:1 and BD groups. In all groups, no significant differences in LDL cholesterol were observed between 0 week and 1 week, but a significant decrease occurred between 1 week and 2 weeks. In the KD 4:1 and KD 1.7:1 groups, no significant changes in HDL cholesterol were recorded across the intervention period. In the BD group, however, the only significant change in HDL cholesterol was a decrease recorded between 1 week and 2 weeks (Table 5).

### 3.5. Changes in Blood Ketone Bodies

**Figure 2:**
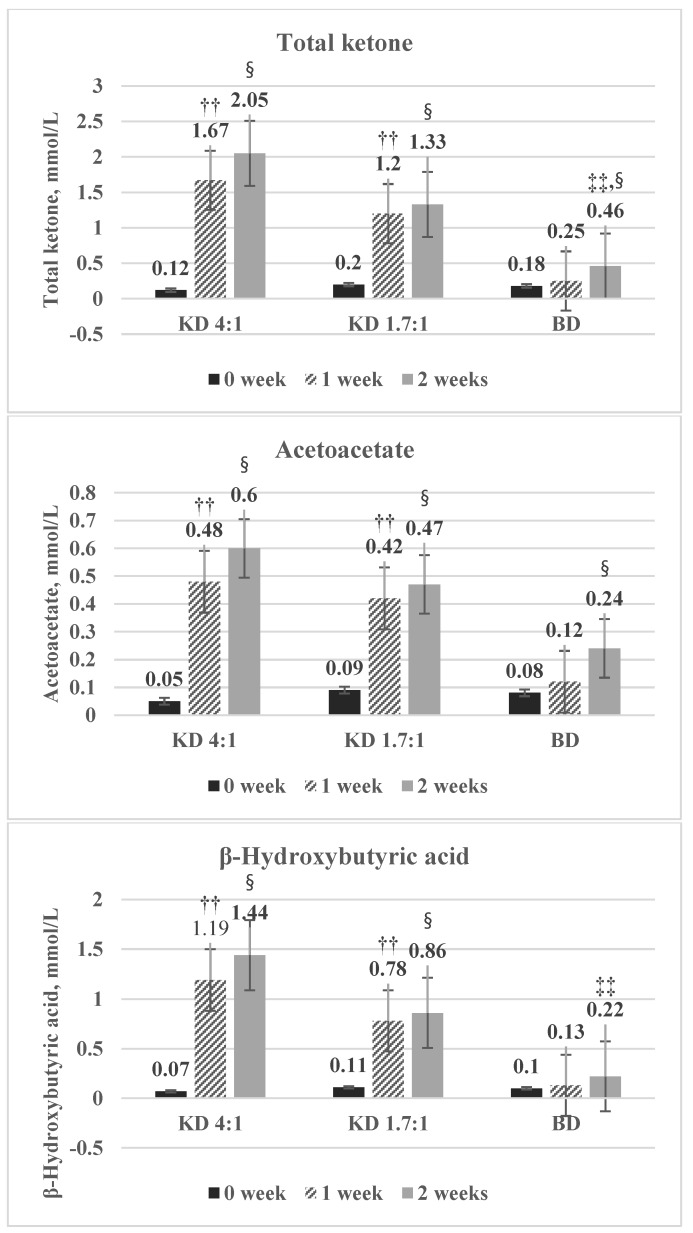
Changes in ketone body biomarkers of each group at 0 week, 1 week, and 2 weeks. ^††^ Significant difference between 0 week and 1 week by Paired *t*-test, Wilcoxon signed rank test. ^‡‡^ Significant difference between 1 week and 2 weeks by Paired *t*-test, Wilcoxon signed rank test. ^§^ Significant difference between 0 week and 2 weeks by Paired *t*-test, Wilcoxon signed rank test.

Total ketone, acetoacetate, and β-Hydroxybutyric acid were significantly increased in the KD4:1 and KD1.7:1 groups at 0 week and 1 week, but there was no significant difference between 1 week and 2 weeks (Figure 2).

### 3.6. Changes in Body Symptoms

**Figure 3:**
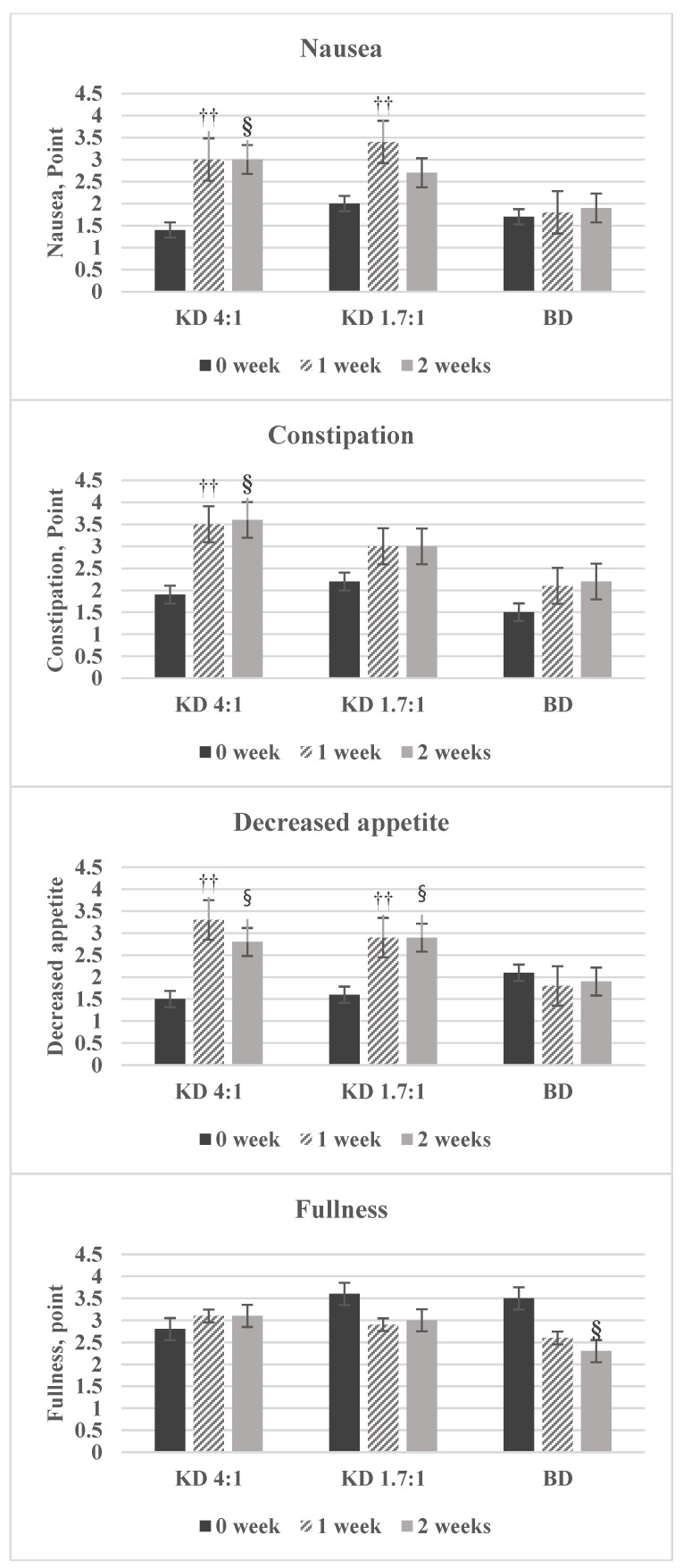
Changes in symptom scores (side effects) reported by each group. ^††^ Significant difference between 0 week and 1 week by Paired *t*-test, Wilcoxon signed rank test; ^§^ Significant difference between 0 week and 2 weeks by Paired *t*-test, Wilcoxon signed rank test.

The symptoms of nausea was significantly increased in the KD4:1 and KD1.7:1 groups at 0 week and 1 week, but there was no significant difference between 1 week and 2 weeks. The symptoms of constipation was significantly increased in the KD4:1 group at 0 week and 1 week. The symptoms of decreased appetite was significantly increased in the KD4:1 and KD1.7:1 groups at 0 week and 1 week, but there was no significant difference between 1 week and 2 weeks. The symptoms of fullness showed no significant change between 0 week and 2 weeks in the KD 4:1 and KD1.7:1 groups, but a significant decrease was recorded in the BD group (Figure 3).

## 4. Discussion

In this study, a two-week comparison of a ketone-to-non-ketone ratio of 4:1 (KD 4:1), modified ketone-to-non-ketone ratio of 1.7:1 (KD 1.7:1), or a balanced nutrition drink (BD) with energy ratio based on Korean nutritional intake standard was performed. Changes in body weight, body composition, blood lipid profiles, blood ketone bodies, and body symptoms were assessed in healthy obese adults. 

In general, a ketogenic diet depletes the glycogen stored in the liver and induces losses of body water and body fat through various complex mechanisms. When carbohydrate intake is limited, the body begins using glycogen to maintain blood glucose levels. If such limited carbohydrate intake is maintained, the majority of the glycogen will be depleted, and lipolysis will increase, and ketone bodies will begin to be generated for energy production. Ketone bodies generated in this way replace glucose as an energy source [24]. Body water reduction occurs because the storage of 1 g of glycogen requires binding with 2 to 4 g of water, and ketone bodies cause a diuretic effect that results in the increased excretion of sodium and water [25]. Low-carbohydrate diet therapy has been reported to reduce body fat and maintain fat-free mass, which is partially a response to the decrease in plasma insulin [16].

Of particular note, it has been reported that weight loss induced in this way does not cause any loss of skeletal muscle. According to a study by Bazzano et al. [14], low-carbohydrate diet therapy in obese adults significantly decreases body fat mass while significantly increasing skeletal muscle mass. Moreno et al. [15] reported that the ketogenic diet applied to obese adults significantly decreases body fat mass while maintaining skeletal muscle mass. In our study, the weight and body fat mass of the subjects declined significantly in all groups between 0 and 1 week and between 1 and 2 weeks, while protein content and skeletal muscle mass remained unchanged only in the KD 1.7:1 group. Body water and minerals showed significant decreases in all groups between 0 week and 1 week but did not change significantly between 1 week and 2 weeks. Additionally, the carbohydrate-protein-fat ratios of the nutrition drinks were 3:7:90 for the KD 4:1 group, 4:16:80 for the KD 1.7:1 group, and 54:16:30 for the BD group, with the protein content being higher for the KD 1.7:1 group than the KD 4:1 group and the energy ratio of protein being in the range of 7% to 20% AMDR of the 2015 KDRIs. The weight loss effect of the ketogenic diet based on ketogenic nutrition drinks with moderate ketogenic ratios is mainly thought to result from body fat reduction, rather than reductions in body water, minerals, and skeletal muscle mass, and that appropriate protein intake is needed to ensure healthy weight loss, where skeletal muscle mass is maintained and only body fat mass decreases. For a ketogenic diet, it is necessary to limit energy intake to a certain degree to maintain ketosis. This has prompted several researchers to claim that the weight loss effect of the diet is not due to the difference in the ratio of carbohydrates, fat, and protein but simply due to the reduction of total energy intake [26,27]. To test for this, we set the same calorie limit for all subject groups in our study.

In this study, the energy intake of all groups was set at 70% of the daily requirement for weight loss, aiming to achieve a caloric deficit of 600 kcal. This was calculated based on treatment guidelines suggesting that low-calorie diet therapies that reduce the intake of calories by about 500 to 1000 kcal a day lead to safe weight loss [28,29]. The nutrition intervention consisted of the consumption of nutrition drinks with different ratios of macronutrients over a two-week period. It is assumed that the observed calorie reduction exceeded the target calorie reduction because of decreased appetite caused by the appetite-suppressing effect of blood ketone bodies and the fact that the subjects consumed only nutrition drinks during the study period. However, the average differences between the daily energy requirement and observed energy intake during the intervention period for each group did not show any significant variation. Therefore, it is expected that the study results can be explained as the effects of the ratio of macronutrients rather than calorie restriction.

The ketogenic diet likely suppresses appetite by reducing the appetite-regulating hormones ghrelin and leptin [30]. Boden et al. [31] reported that low-carbohydrate diets cause significant decreases in ghrelin and leptin, and Johnstone et al. [32] found that hunger was significantly suppressed among subjects following the ketogenic diet. Regarding changes in body symptoms during the study period, appetite decreased significantly between 0 week and 1 week in the KD 4:1 and KD 1.7:1 groups and remained largely unchanged between 1 week and 2 weeks. Fullness was significantly different in BD according to time. Appetite reduction and fullness maintenance were observed only in the KD 4:1 and KD 1.7:1 groups, despite the fact that all three groups consumed nutrition drinks only, meaning that this effect can likely be explained by an increase in appetite-regulating hormones.

The ketogenic diet has a significantly positive effect in that it decreases total cholesterol and LDL cholesterol and increases HDL cholesterol [17,18,19]. In general, it has direct, diet-related effects on overall endogenous cholesterol synthesis. The core enzyme of cholesterol biosynthesis is 3-hydroxy-3-methylglutaryl CoA reductase, which is activated by insulin. If the insulin level increases due to increased blood glucose, endogenous cholesterol synthesis increases as well. The restriction of carbohydrate intake through the ketogenic diet creates the opposite effect. In addition, ketosis can improve the blood lipid profile, suggesting beneficial effects on the risk factors for cardiovascular disease [11,18]. According to a study by Moreno et al. [15], the ketogenic diet causes a significant reduction in total cholesterol in obese adults. Hu et al. [18] reported significant decreases in total cholesterol, triglycerides, and LDL cholesterol but a notable increase in HDL cholesterol. Phytosterols in plant oils and nuts have structures similar to that of cholesterol and thus compete with cholesterols in the body, leading to the inhibition of cholesterol absorption and a decrease in serum cholesterol [33]. Kris-Etherton et al. [34] reported that, in a group following a diet high in mono-unsaturated fatty acids, total cholesterol decreased by 10% and LDL cholesterol decreased by 14%. 

In the KD 1.7:1 and BD groups, total cholesterol and LDL cholesterol decreased significantly between 0 week and 2 weeks. In all groups, HDL cholesterol showed no significant change between 0 week and 2 weeks, but decreased significantly in the BD group between 1 week and 2 weeks. The KD 4:1 and KD 1.7:1 groups produced different results in terms of blood lipid profile changes, showing that increases in fat content or ketogenic ratio do not always lead to improvement in the blood lipid profile, despite the intake of fat mainly in the form of unsaturated fatty acids. 

Although there is no standardized ketogenic diet therapy, it can be classified as a diet with a carbohydrate intake of less than 10% or a total daily carbohydrate intake of less than 30 g [23]. Therefore, further study on the effect of carbohydrate, protein, fat energy ratio or ketogenic ratio on obese adults is needed. When a diet with a ketone-to-non-ketone ratio of at least 1.5:1 is consumed, the concentration of ketone bodies in the blood and urine increase. In physiological ketosis caused by the low-calorie ketogenic diet or fasting, ketonemia has a maximum level of 7 to 8 mmol/L without pH changes. The concentration of ketone bodies rarely exceeds 8 mmol/L, because the central nervous system (CNS) uses ketone bodies as an energy source instead of glucose quite quickly. After two weeks of the intervention, the total ketone concentration was 2.05 ± 1.55 mmol/L in the KD 4:1 group, 1.33 ± 0.85 mmol/L in the KD 1.7:1 group, and 0.46 ± 0.36 mmol/L in the BD group, none of which exceeded 8 mmol/L. Additionally, in the KD 4:1 and KD 1.7:1 groups, total ketone, acetoacetate, and β-hydroxybutyric acid levels increased significantly between 0 week and 1 week but then remained steady between 1 week and 2 week. It thus seems that the consumption of ketogenic nutrition drinks with a ketogenic ratio of either 4:1 or 1.7:1 is sufficient to induce ketosis, which can then be maintained without increasing the ratios further, as the ketone production capability of the liver is limited and ketone bodies are used as an energy source by other tissues.

The strength of this study lies on the use of nutrition drinks, which allowed for accurate estimation of energy and nutrient intake and thereby keeping track of accurate ketogenic ratios. In typical food intake-type studies, the amounts of foods consumed are determined through the 24-h recall method or self-reporting method, which limit accuracy due to their reliance on the memory of the study participants. Additionally, in low-carbohydrate diets for Asians, choices of food ingredients are quite limited, as many of the staple foods in Asian countries are high in carbohydrates. Moreover, cooking can be difficult due to poor emulsification caused by the higher fat ratios. These limitations in the preparation and maintenance of ketogenic diets were overcome in this study through the easy use of liquid-type nutrition drinks. Second, the study was monitored through questionnaires to ensure that the study participants maintained their usual levels of physical activity, and the consumption of other foods besides the nutrition drinks was monitored using urinary ketone body test strips, allowing for the accurate measurement of the effects of the nutritional intervention.

This study however, has a few limitations. First, the long-term effects and safety of this study could not be determined because it was a short-term intervention that spanned a period of only two weeks, making it difficult to recommend it for general people as a diet therapy for health improvement [35]. Second, although the ketogenic diet used in this study produced some positive results in terms of weight loss and blood lipid profile improvement, compliance was rather low, and the drop-out rate was high at 50% for KD4:1 group and 38.9% for KD1.7:1 group. It is assumed that the low compliance was likely a result of the liquid-type diet used. However, considering that the KD1.7:1 group showed similar drop-out rate (32.8%, 34.1%) compared to the previous studies [15,19], we could speculate that maintaining 4:1 ratio of ketogenic diet is rather a very harsh regimen to follow. Thus, as this study observed similar effects for a ketogenic diet with the typical ketogenic ratio of 4:1 and a ketogenic diet with a more moderate ketogenic ratio of 1.7:1, it believed that more studies on diets involving general meals or general meals combined with ketogenic nutrition drinks with nutrient compositions similar to those of the drinks developed in this study need to be conducted.

## 5. Conclusions

This study is meaningful as a nutritional intervention clinical trial that investigated changes in body weight, body composition, blood lipid profile, and blood ketone bodies. We evaluated three groups of subjects after two weeks of consuming of one of two ketogenic nutrition drinks with different ketogenic ratios or a balanced nutrition drink, all of which provided the same amount of energy below the daily energy requirement for weight loss. In conclusion, the production of ketone bodies was induced and maintained through consumption of a ketogenic nutrition drink with a more moderate ketogenic ratio (1.7:1) than the typical ratio of 4:1 that was also used in this study and has been used in other similar studies. This study showed that a ketogenic diet that induces ketosis through the consumption of a ketogenic nutrition drink can maintain skeletal muscle mass while reducing body fat mass. Although blood lipid profiles were improved, the improvements of blood lipid values differed with the ketogenic ratio. Thus, it is clear that more studies on the effects of this diet that take into account the energy ratio of carbohydrates, protein, and fat and the ketogenic ratio in obese adults need to be conducted in the future. This study also showed the appetite-reduction and fullness maintenance effects of two ketogenic nutrition drinks with different ketogenic ratios, indicating the potential for development of ketogenic nutrition drinks or diets based on the nutrient composition used in this study for the treatment of obesity through customization to individual characteristics and medical conditions. Furthermore, as this intervention succeeded in inducing and maintaining ketosis through the development and use of a ketogenic nutrition drink with a moderate ketogenic ratio of 1.7, it is possible that the results of this study may be used in the development of adjuvant therapies for other diseases.

## Figures and Tables

**Table 1:** Composition of nutrition drinks.

	KD4:1 ^(1)^	KD1.7:1 ^(2)^	BD ^(3)^
Energy (kcal)	200	200	200
C:P:F (% of kcal) ^(4)^	3:7:90	4:16:80	54:16:30
Carbohydrate (g) ^(5)^	1.7	2.3	28.0
Fiber (g) ^(6)^	0.0	0.7	1.0
Protein (g) ^(7)^	3.3	8.0	8.0
Fat (g) ^(8)^	20.0	17.7	7.0
Saturated fat (g) ^(9)^	4.2	0.0	2.0

^(1)^ KD4:1 = Ketogenic nutrition drink 4:1 (Ketonina, Namyang Dairy Product Co Ltd., Seoul, Korea). ^(2)^ KD1.7:1 = Ketogenic nutrition drink 1.7:1 (Product for research, Maeil Dairies Co Ltd., Gyeonggi-doKorea). ^(3)^ BD = Balanced nutrition drink (Mediwell Nutty flavor, Maeil Dairies Co Ltd., Gyeonggi-do, Korea). ^(4)^ C:P:F = Carbohydrate:Protein:Fat (% of kcal). ^(5)^ KD4:1 = Maltodextrin, white sugar; KD1.7:1 = Maltodextrin, white sugar; BD = Maltodextrin, white sugar. ^(6)^ KD4:1 = Soybean fiber; KD1.7:1 = Soybean fiber, non-digestible maltodextrin/indigestible maltodextrin; BD = Soybean fiber. ^(7)^ KD4:1 = Sodium caseinate, milk fat globule membrane protein; KD1.7:1 = Sodium caseinate, isolated soy protein; BD = Sodium caseinate, isolated soy protein. ^(8)^ KD4:1 = Pure olive oil (extra virgin olive oil, δ tocopherol (mixed)), edible oils and fats (soybean oil, high oleic sunflower oil, coconut oil, δ tocopherol (mixed)), canola oil; KD1.7:1 = High oleic sunflower oil (3.7%), canola oil (3.7%); BD = Canola oil (3%). ^(9)^ KD4:1 = Medium Chain Triglyceride (MCT) oil; KD1.7:1 = MCT oil (3.3%); BD = MCT oil (0.006%).

**Table 2:** Baseline characteristics of the study participants.

Variables	KD4:1 ^(3)^ (*n* = 13)	KD1.7:1 ^(4)^ (*n* = 13)	BD ^(5)^ (*n* = 11)	*p*-Value ^(9)^
Age (years)	29.5 ± 9.0 ^(1)^	24.5 ± 4.8	26.0 ± 7.5	0.141
Sex (%)				
Female	4 (30.8) ^(2)^	5 (38.5)	4 (36.4)	0.739
Male	9 (69.2)	8 (61.5)	7 (63.6)	
Height (cm)	170.3 ± 12.4	169.9 ± 10.0	171.1 ± 7.6	0.962
Body composition				
Body water (L)	41.8 ± 8.9	41.1 ± 9.1	41.7 ± 7.9	0.974
Protein (kg)	11.3 ± 2.5	11.2 ± 2.5	11.3 ± 2.2	0.975
Mineral (kg)	4.0 ± 0.9	4.0 ± 0.9	4.0 ± 0.7	0.998
Body weight (kg)	87.3 ± 17.1	82.9 ± 14.3	85.8 ± 14.8	0.764
Skeletal muscle (kg)	32.2 ± 7.4	31.6 ± 7.6	32.1 ± 6.5	0.974
Body fat mass (kg)	30.2 ± 10.1	26.7 ± 7.3	28.8 ± 7.6	0.862
BMI (kg/m^2^) ^(6)^	30.0 ± 4.2	28.5 ± 2.8	29.2 ± 4.0	0.779
Waist circumference (cm)	100.0 ± 11.3	94.2 ± 7.3	96.3 ± 10.1	0.318
Hip circumference (cm)	107.9 ± 7.5	108.6 ± 6.2	108.7 ± 7.2	0.950
Blood lipid profile				
Total cholesterol (mg/dL)	184.9 ± 31.2	179.8 ± 30.2	182.8 ± 28.9	0.912
Triglyceride (mg/dL)	142.4 ± 69.6	106.2 ± 45.8	101.5 ± 63.4	0.218
LDL cholesterol (mg/dL) ^(7)^	116.2 ± 28.4	111.4 ± 31.3	120.3 ± 23.3	0.742
HDL cholesterol (mg/dL) ^(8)^	50.8 ± 9.5	57.9 ± 11.7	52.6 ± 16.0	0.331

^(1)^ Values are means ± SD; ^(2)^ Values are *N* (%); ^(3)^ KD4:1 = Ketogenic nutrition drink 4:1; ^(4)^ KD1.7:1 = Ketogenic nutrition drink 1.7:1; ^(5)^ BD = Balanced nutrition drink; ^(6)^ BMI = Body mass index; ^(7)^ LDL = Low-density lipoprotein; ^(8)^ HDL = High-density lipoprotein; ^(9)^ Significantly different at *p* < 0.05 by chi-square test, Fisher’s exact test, ANOVA test, or Kruskal–Wallis test.

**Table 3:** Daily dietary intake in each group during two weeks of intervention.

	KD4:1 ^(2)^ (*n* = 8)	KD1.7:1 ^(3)^ (*n* = 11)	BD ^(4)^ (*n* = 11)	*p*-Value ^(6)^
Energy (kcal)	1159.1 ± 416.1 ^(1)^	1280.8 ± 262.2	1357.1 ± 292.5	0.583
Energy deficit ^(5)^	799.7 ± 373.2	743.8 ± 157.5	716.2 ± 94.4	0.765
Carbohydrate (g)	9.7 ± 3.5 ^c^	14.5 ± 3.0 ^b^	190.0 ± 40.9 ^a^	0.000
Fiber (g)	0.0 ± 0.0 ^c^	4.3 ± 0.9 ^b^	6.8 ± 1.5 ^a^	0.000
Protein (g)	19.3 ± 6.9 ^b^	51.2 ± 10.5 ^a^	54.3 ± 11.7 ^a^	0.000
Fat (g)	115.9 ± 41.6 ^a^	113.6 ± 23.2 ^a^	47.5 ± 10.2 ^b^	0.000
Saturated fat (g)	24.1 ± 8.7 ^a^	0.0 ± 0.0 ^c^	13.6 ± 2.9 ^b^	0.000

^(1)^ Values are means ± SD; ^(2)^ KD4:1 = Ketogenic nutrition drink 4:1; ^(3)^ KD1.7:1 = Ketogenic nutrition drink 1.7:1; ^(4)^ BD = Balanced nutrition drink; ^(5)^ Deficit of energy requirements and energy intake; ^(6)^ Values with different letters in a column indicate statistically significant difference using Kruskal–Wallis test by Mann–Whitney U test (*p* < 0.05 ).

**Table 4:** Changes in anthropometry and body composition by each group.

	KD4:1 ^(2)^			KD1.7:1 ^(3)^			BD ^(4)^		
	Δ1 week-0 week ^(5)^ (*n* = 13)	Δ2 week-1 week ^(6)^ (*n* = 8)	Δ2 week-0 week ^(7)^ (*n* = 8)	Δ1 week-0 week ^(5)^ (*n* = 13)	Δ2 week-1 week ^(6)^ (*n* = 11)	Δ2 week-0 week ^(7)^ (*n* = 11)	Δ1 week-0 week ^(5)^ (*n* = 11)	Δ2 week-1 week ^(6)^ (*n* = 11)	Δ2 week-0 week ^(7)^ (*n* = 11)
Body water (L)	−2.2 ± 1.3 ^∫(1)^	−0.5 ± 0.8	−2.8 ± 1.1 ^∫^	−1.1 ± 1.4 ^†^	−0.3 ± 1.0	−1.5 ± 1.1 ^‡^	−1.2 ± 0.7 ^∫^	−0.3 ± 0.5	−1.5 ± 0.8 ^∫^
Protein (kg)	−0.5 ± 0.3 ^∫^	−0.2 ± 0.2 ^†^	−0.7 ± 0.3 ^∫^	−0.3 ± 0.4	−0.1 ± 0.3	−0.4 ± 0.3 ^‡^	−0.3 ± 0.2 ^∫^	−0.1 ± 0.2 ^†^	−0.4 ± 0.2 ^∫^
Mineral (kg)	−0.2 ± 0.1 ^∫^	−0.0 ± 0.1	−0.3 ± 0.1 ^‡^	−0.1 ± 0.1 ^†^	−0.0 ± 0.1	−0.2 ± 0.1 ^∫^	−0.1 ± 0.1 ^∫^	−0.1 ± 0.1	−0.2 ± 0.1 ^‡^
Body weight (kg)	−3.9 ± 1.4 ^∫^	−1.7 ± 0.7 ^∫^	−5.9 ± 1.8 ^∫^	−3.3 ± 1.2 ^∫^	−1.0 ± 1.1 ^†^	−4.4 ± 1.5 ^∫^	−2.3 ± 1.0 ^∫^	−1.1 ± 0.7 ^∫^	−3.4 ± 1.3 ^∫^
Skeletal muscle (kg)	−1.5 ± 1.0 ^†^	−0.4 ± 0.5 ^†^	−2.1 ± 0.8 ^∫^	−0.7 ± 1.2	−0.3 ± 0.8	−1.0 ± 1.0 ^‡^	−0.9 ± 0.5 ^∫^	−0.3 ± 0.4 ^†^	−1.2 ± 0.6 ^∫^
Body fat mass (kg)	−1.1 ± 1.0 ^†^	−1.0 ± 0.8 ^‡^	−2.2 ± 1.4 ^∫^	−1.8 ± 2.0 ^‡^	−0.5 ± 0.8 ^†^	−2.5 ± 1.9 ^‡^	−0.8 ± 0.5 ^‡^	−0.6 ± 0.6 ^†^	−1.3 ± 0.7 ^‡^
BMI (kg/m^2^) ^(8)^	−1.4 ± 0.5 ^∫^	−0.5 ± 0.2 ^∫^	−2.1 ± 0.6 ^†^	−1.1 ± 0.4 ^∫^	−0.3 ± 0.4 ^†^	−1.5 ± 0.5 ^∫^	−0.8 ± 0.3 ^‡^	−0.4 ± 0.3 ^‡^	−1.2 ± 0.4 ^‡^
Waist circumference (cm)	−3.5 ± 2.5 ^∫^	−1.4 ± 1.7	−4.8 ± 2.1 ^∫^	−3.7 ± 2.6 ^∫^	−0.8 ± 2.2	−4.6 ± 3.9 ^‡^	−3.0 ± 1.9 ^‡^	−1.5 ± 1.7 ^†^	−4.5 ± 2.0 ^‡^
Hip circumference (cm)	−2.0 ± 1.8 ^‡^	−2.0 ± 1.5 ^‡^	−3.9 ± 2.0 ^‡^	−1.8 ± 1.8 ^‡^	−1.1 ± 0.9 ^‡^	−3.1 ± 1.0 ^∫^	−1.2 ± 0.8 ^‡^	−0.9 ± 1.5	−2.1 ± 1.6 ^‡^

^(1)^ Values are means ± SD, Significance as determined by Paired t-test, Wilcoxon signed rank test. *p*-value less than ^†^ *p* < 0.05, ^‡^ *p* < 0.01, ^∫^ *p* < 0.001.; ^(2)^ KD4:1 = Ketogenic nutrition drink 4:1; ^(3)^ KD1.7:1 = Ketogenic nutrition drink 1.7:1; ^(4)^ BD = Balanced nutrition drink; ^(5)^ Difference between 1 week and 0 week; ^(6)^ Difference between 2 weeks and 1 week; ^(7)^ Difference between 2 weeks and 0 week; ^(8)^ BMI = Body mass index.

**Table 5:** Changes in blood lipids by group.

	KD4:1 ^(2)^			KD1.7:1 ^(3)^			**BD ^(4)^**		
	Δ1 week-0 week ^(5)^ (*n* = 13)	Δ2 week-1 week ^(6)^ (*n* = 8)	Δ2 week-0 week ^(7)^ (*n* = 8)	Δ1 week-0 week ^(5)^ (*n* = 13)	Δ2 week-1 week ^(6)^ (*n* = 11)	Δ2 week-0 week ^(7)^ (*n* = 11)	**Δ1 week-0 week ^(5)^ (*n* = 11)**	**Δ2 week-1 week ^(6)^ (*n* = 11)**	**Δ2 week-0 week ^(7)^ (*n* = 11)**
Total cholesterol (mg/dL)	2.5 ± 21.9 ^(1)^	−23.0 ± 15.1 ^‡^	−18.3 ± 22.0	1.3 ± 18.9	−18.9 ± 18.2 ^‡^	−14.0 ± 13.9 ^‡^	2.6 ± 16.0	−14.6 ± 17.2 ^†^	−11.9 ± 15.2 ^†^
Triglyceride (mg/dL)	−18.4 ± 99.9	7.4 ± 57.8	−27.7 ± 43.9	−7.9 ± 44.3	−5.0 ± 30.1	−14.3 ± 33.9	−8.4 ± 44.6	7.2 ± 22.6	−1.2 ± 40.9
LDL cholesterol (mg/dL) ^(8)^	8.2 ± 20.6	−24.5 ± 15.3 ^‡^	−11.6 ± 22.6	3.4 ± 17.4	−15.5 ± 18.2 ^†^	−9.6 ± 13.2 ^†^	4.6 ± 13.9	−15.0 ± 14.8 ^‡^	−10.4 ± 9.2 ^‡^
HDL cholesterol (mg/dL) ^(9)^	−1.5 ± 10.6	0.6 ± 6.8	−0.6 ± 13.7	−2.8 ± 5.9	−0.1 ± 3.8	−2.3 ± 3.8	−0.8 ± 6.8	−2.9 ± 3.2 ^†^	−3.7 ± 8.2

^(1)^ Values are means ± SD, Significance as determined by Paired *t*-test, Wilcoxon signed rank test. *p*-value less than ^†^ *p* < 0.05, ^‡^ *p* < 0.01; ^(2)^ KD4:1 = Ketogenic nutrition drink 4:1; ^(3)^ KD1.7:1 = Ketogenic nutrition drink 1.7:1; ^(4)^ BD = Balanced nutrition drink; ^(5)^ Difference between 1 week and 0 week; ^(6)^ Difference between 2 weeks and 1 week; ^(7)^ Difference between 2 weeks and 0 week; ^(8)^ LDL = Low density lipoprotein; ^(9)^ HDL = High density lipoprotein.
